# Jascayd (nerandomilast): a novel PDE4B inhibitor for idiopathic pulmonary fibrosis

**DOI:** 10.1097/MS9.0000000000004290

**Published:** 2025-11-08

**Authors:** Syeda Fadak Zahra Hujjat, Harshika Khaim Chandani, Devya Khaim Chandani, Muhammad Saad Khan, Abubakr Mahmoud

**Affiliations:** a Department of medicine, Allama Iqbal Medical College, Lahore, Pakistan; b Department of Medicine, Jinnah Sindh Medical University, Karachi, Pakistan; c Department of medicine, Shaheed Mohtarma Benazir Bhutto Medical College (SMBBMC), Karachi, Pakistan; d Department of Medicine, University of Khartoum, Khartoum, Sudan

**Keywords:** antifibrotic therapy, idiopathic pulmonary fibrosis, Jascayd, nerandomilast, PDE4B inhibitor

## Abstract

Idiopathic pulmonary fibrosis (IPF) is a progressive interstitial pneumonia with poor prognosis and limited therapeutic options. Despite the introduction of pirfenidone and nintedanib, treatment discontinuation due to adverse effects remains common, highlighting the need for better-tolerated, mechanistically distinct agents. Jascayd (nerandomilast), a first-in-class selective phosphodiesterase-4B inhibitor developed by Boehringer Ingelheim, was approved by the FDA in October 2025 for adults with IPF. By elevating intracellular cyclic adenosine monophosphate (cAMP) in inflammatory and structural lung cells, nerandomilast suppresses pro-inflammatory cytokines and fibroblast activation, producing anti-inflammatory and antifibrotic effects with improved tolerability over nonselective PDE4 inhibitors. In phase 3 FIBRONEER-IPF and FIBRONEER-ILD trials, nerandomilast significantly reduced the annual rate of forced vital capacity decline versus placebo, regardless of concomitant antifibrotic use. Common adverse effects included mild diarrhea, nausea, and fatigue, with low discontinuation rates. Long-term extension data demonstrate sustained safety and lung function preservation. Offering both monotherapy and combination potential, Jascayd establishes a new therapeutic class that targets inflammation and fibrosis concurrently, representing a pivotal advancement toward personalized, multi-pathway treatment in IPF.

## Background

Idiopathic pulmonary fibrosis (IPF), an unidentified cause of persistent and progressive fibrosing interstitial pneumonia, is distinguished by its histopathological typical interstitial pneumonia pattern. It mainly affects elderly persons and is linked to a median survival of just 3–5 years increasing dyspnea and diminishing lung function^[1]^. The incidence is increasing as a result of better diagnosis and aging populations and the global prevalence is between 10 and 60 per 100 000^[2]^. Prior to 2014 the oxygen therapy, pulmonary rehabilitation and lung transplantation were the mainstays of supportive care. Although they provided only slight benefits in the reduction of forced vital capacity (FVC), the approvals of pirfenidone and nintedanib introduced the first disease-modifying alternatives^[3]^. Because of side effects such weariness, nausea, or liver toxicity, many patients stop taking these treatments^[4]^.

**HIGHLIGHTS**

- **First-in-Class Mechanism**: Jascayd is the first selective phosphodiesterase-4B (PDE4B) inhibitor approved for idiopathic pulmonary fibrosis (IPF), offering a novel anti-inflammatory and antifibrotic approach distinct from existing therapies.
- **Proven Efficacy in Pivotal Trials**: The FIBRONEER phase 3 program demonstrated that Jascayd significantly slows disease progression by reducing the rate of forced vital capacity decline.
- **Improved Tolerability Profile**: Its selectivity for the PDE4B subtype results in a significantly lower incidence of nausea and psychiatric side effects compared to older, non-selective PDE4 inhibitors.
- **Flexible Treatment Regimen**: Jascayd can be administered as a convenient twice-daily oral tablet, either as monotherapy or in combination with pirfenidone or nintedanib, without a significant increase in adverse events.
- **Advances IPF Management**: It represents a significant therapeutic advancement, providing a new, well-tolerated option to slow functional decline and introducing a potential for multi-pathway combination therapy.

PDE4 inhibition, a mechanism linked to inflammation and fibroblast activation, became the focus of study once it was realized that better-tolerated mechanistically unique treatments were needed. Jascayd (nerandomilast), created by Boehringer Ingelheim, was approved by the FDA on 10 October 2025 as the first-in-class selective phosphodiesterase-4B (PDE4B) inhibitor for adult patients with IPF^[5]^. This approval came after nerandomilast’s safety and effectiveness in reducing the progression of the disease were validated by the results of two critical global phase 3 studies. This manuscript has been developed in accordance with the TITAN Guidelines (2025)^[6]^.

## Mechanism of action and pharmacokinetics

The PDE4B isoform, which hydrolyzes intracellular cAMP in inflammatory and structural lung cells, is specifically inhibited by nerandomilast. Nerandomilast inhibits fibroblast proliferation and myofibroblast differentiation while suppressing the release of pro-inflammatory cytokines such TNF-α, IL-6, and IL-1β by raising intracellular cAMP^[7]^.

PDE4B specificity enables anti-inflammatory and antifibrotic effects with better tolerability than previous nonselective PDE4 inhibitors that inhibited all PDE4 isoforms and produced unbearable nausea. Pharmacokinetically, it is quickly absorbed when taken orally and reaches steady-state levels in a few days. It supports twice-daily oral administration due to its half-life of roughly 12–14 hours. Hepatic oxidation is the main mode of metabolism with metabolites being excreted by the kidneys and feces^[8]^. In both healthy and IPF participants, the medication has predictable linear kinetics and little buildup.

## Dosing and administration

Jascayd should be taken orally twice a day at a dose of 18 mg, either with or without food. Either monotherapy or combination with approved antifibrotics (pirfenidone or nintedanib) can be used to start treatment. Patients in the FIBRONEER trials continued their antifibrotic medication without experiencing any more side effects, indicating compatibility and potential for combination. Every 12–24 weeks routine monitoring consists of lung function evaluation, gastrointestinal tolerance assessment, and liver function testing. Patients who have severe nausea or chronic diarrhea may benefit from dose decreases. For mild-to-severe renal or hepatic impairment, there are currently no guidelines for dose adjustments. Pregnancy, lactation, and severe liver impairment have not been proven to be safe^[9]^.

## Evaluation of safety and efficacy through clinical trials

Over a 52-week period, nerandomilast 18 mg twice daily was compared to a placebo in the FIBRONEER-IPF phase 3 trial (*N* = 1000)^[10]^. Rate of reduction in FVC, the main endpoint, was significantly attenuated in the nerandomilast arm (–70 mL) as opposed to the placebo (–154 mL; *P* < 0.01). This effect held true irrespective of the antifibrotic medication that was previously administered.

Similar findings were obtained by the FIBRONEER-ILD trial, which assessed nerandomilast in progressive pulmonary fibrosis of non-IPF origin demonstrating wide antifibrotic activity. Diarrhea was the most frequent adverse event (34–36%), usually mild to moderate, and less than 10% of cases resulted in discontinuation. Headache, exhaustion, and nausea were other adverse effects^[11]^.

The FIBRONEER-ON open-label extension experiment, which included patients who finished the FIBRONEER-IPF and FIBRONEER-ILD investigations, is further assessing the long-term safety and effectiveness of nerandomilast. Over the course of about 96 weeks of continuous medication, the trial seeks to evaluate lung function preservation, adverse events, and sustained tolerability. Nerandomilast is administered to all enrolled patients, whether or not they are receiving prior antifibrotic treatment. The drug’s potential for long-term usage is supported by interim analyses that have so far shown a consistent safety profile free of cumulative or novel toxicities^[12]^.

Crucially, there were no new safety signals, hepatotoxicity, or mental problems, and the safety profile was the same for both the monotherapy and combination groups. Data from long-term extensions demonstrate consistent lung function preservation over 96 weeks and constant tolerability^[13]^.

## Comparative perspective

By inhibiting PDE4B, nerandomilast targets inflammation and fibroblast proliferation in contrast to pirfenidone and nintedanib that block TGF-β-mediated and tyrosine kinase-driven fibrogenic signaling, respectively^[14]^. Its distinct mechanism enhances the effects of the current medications, opening the possibility of combination therapy for further benefits. Additionally, the nausea, weight loss, and mental health issues linked to previous PDE4 inhibitors like roflumilast are significantly lessened by nerandomilast’s selective profile^[7]^. One of the main drawbacks of earlier antifibrotics was adherence, which this tolerance may improve. There are not any head-to-head comparisons, but when used either alone or in combination, FVC preservation with nerandomilast seems to be on par with or better than existing standards. From a therapeutic perspective, the release of Jascayd (nerandomilast) is set to transform therapy protocols for IPF. It allows for earlier intervention and personalized treatment by offering a more pleasant orally taken alternative with both anti-inflammatory and antifibrotic effects. Clinicians might contemplate its application in patients who are intolerant to current antifibrotics or as a supplementary treatment in instances of rapid functional deterioration. Importantly, Jascayd’s good safety record could help people stick with it for a long time, which is a big problem with current treatments.

## Complications

Diarrhea is the most common adverse event, typically occurring in the first 2 months and affecting around one-third of patients receiving treatment^[12]^. Fatigue, nausea, and abdominal discomfort are some typical effects. There have been isolated reports of hypersensitivity and elevated liver enzymes. Though they were not shown in large trials, weight loss and mood swings are possible hazards associated with PDE4 inhibitors^[8]^. Dehydration and gastric tolerance should be closely watched in patients. In general side effects are dose related and controllable by symptomatic treatment or brief dose reductions.

## Cost-effectiveness and future implications

According to economic analyses, even a slight slowdown in the decline of FVC can result in a considerable increase in IPF’s quality-adjusted life year^[3]^. Given the high annual costs of conventional antifibrotics, the price strategy for Jascayd will influence access and health-system sustainability. The drug’s oral twice-daily dose, excellent tolerability, and potential combination use may reduce hospitalization and enhance adherence, thus enhancing cost-effectiveness over time. Future studies should study its usage in earlier phases of IPF and combination regimens as well as biomarker-driven therapy to identify responders^[11]^. The PDE4B pathway may also offer potential across other chronic lung illnesses including chronic hypersensitivity pneumonitis and rheumatoid arthritis-associated Interstitial Lung Disease (ILD). Aside from IPF, the selective PDE4B inhibition signifies a prospective research direction in secondary interstitial lung disorders, including rheumatoid arthritis-associated ILD, systemic sclerosis-related fibrosis, and chronic hypersensitivity pneumonitis. Combination regimens that couple Jascayd with well-known antifibrotics or new drugs that target TGF-β and integrin pathways should be looked into to see whether they can provide extra benefits. Biomarker-guided therapy and earlier commencement in pre-fibrotic stages may enhance results and reframe disease change in fibrotic lung illnesses^[15]^.

## Limitations and contraindications

Nerandomilast does not reverse established fibrosis; it only slows progression. Long-term mortality benefit remains unproven, and further postmarketing surveillance is warranted. Contraindications include hypersensitivity to nerandomilast or any excipient and severe hepatic impairment. Caution is advised in patients with uncontrolled gastrointestinal disorders or psychiatric illness. The safety of the drug in pediatric, pregnant, or lactating populations is unestablished^[13]^.

## Conclusion

As the first-in-class selective PDE4B inhibitor with demonstrated antifibrotic efficacy and good tolerability, Jascayd (nerandomilast) represents a significant advancement in the treatment of IPF. Its ability to maintain lung function across a variety of fibrotic phenotypes along with its compatibility with current antifibrotics represents a new therapeutic paradigm in IPF. Despite ongoing concerns about long-term outcomes and cost, it gives patients with this debilitating disease new hope and opens the door to multi-pathway combination-based treatment approaches that could further revolutionize care. It is not merely an enhancement to the antifibrotic arsenal, but it represents a transition to a multi-pathway and mechanism-based approach to managing IPF, combining the regulation of inflammation with the inhibition of fibrosis. Its tolerability and potential for combination use make it a viable and forward-looking alternative in daily clinical practice.

## Data Availability

No data were generated for this manuscript.

## Writing assistance

None used.
